# Prospective multicenter validation of a machine learning model for predicting anastomotic leakage in patients with gastric adenocarcinoma undergoing total or proximal gastrectomy

**DOI:** 10.1097/JS9.0000000000003025

**Published:** 2025-07-17

**Authors:** Shengli Shao, Yanqi Li, Huangrong Cheng, Chao Chen, Ying Zeng, Wenjun Huang, Haiping Luo, Xiaoming Yu, Xiaoping Yin, Xinmeng Sun, Jichao Qin

**Affiliations:** a Department of Surgery, Tongji Hospital, Tongji Medical College, Huazhong University of Science and Technology, Wuhan, Hubei, China; b The Affiliated Cancer Hospital of Zhengzhou University & Henan Cancer Hospital, Zhengzhou, China; c Department of Gastrointestinal Surgery, Xianning Central Hospital, The First Affiliated Hospital of Hubei University of Science and Technology, Xianning, China; d Department of Gastrointestinal Surgery, the First Affiliated Hospital, Zhejiang University School of Medicine, Hangzhou, China; e Department of Nursing, the First Affiliated Hospital, Zhejiang University School of Medicine, Hangzhou, China; f Zhejiang University, Hangzhou, China; g Department of Gastrointestinal Surgery, Huangshi Central Hospital, Affiliated Hospital of Hubei Polytechnic University, Huangshi, China; h Department of Surgery, Huanggang Central Hospital of Yangtze University, Huanggang, China

**Keywords:** anastomotic leakage, gastrectomy, gastric adenocarcinoma, machine learning model

## Abstract

**Objective:**

Predicting esophago-gastric and esophagojejunal anastomotic leakage (AL) is inherently challenging. The aim of the present study was to investigate the clinical utility of a real-time machine learning model for predicting AL.

**Background:**

AL is one of the most serious postoperative complications following esophagogastric and esophagojejunal anastomoses. Traditional risk stratification methods have often struggled to accurately predict which patients are most at risk, owing to the multifactorial nature of AL and the variability in patient and operative factors.

**Methods:**

In this prospective study, gastric adenocarcinoma patients who were scheduled for total or proximal gastrectomy from four medical centers were enrolled between January 2022 and January 2024. During operations, a developed machine learning model was used to assess the risk of AL. The primary outcome is the occurrence of AL.

**Results:**

A total of 512 patients were included. AL was observed in 13 patients (2.54%). The model yielded an area under the operating characteristic curve of 0.780, a sensitivity of 0.769, a specificity of 0.577 and a negative predictive value of 0.990. Of the 512 patients, 221 were identified as high-risk and 291 as low-risk. Compared with the low-risk group, the AL rate was significantly higher in the high-risk group (10/221 vs. 3/291; *P* = 0.027). Post hoc analysis revealed ~ 35% (risk score<0.45) patients can safely avoid intensive monitoring.

**Conclusions:**

By achieving high sensitivity while excluding nearly half of the non-AL subgroups, the model (https://gasal.21cloudbox.com/) provides effective risk stratification of AL in patients with gastric adenocarcinoma undergoing esophagogastrostomy or esophagojejunostomy.

## Introduction

Gastric adenocarcinoma is the most common malignant tumor in the upper gastrointestinal tract^[1]^. Surgery is a crucial treatment modality for gastric cancer, with total and proximal gastrectomy being the primary surgical procedures for tumors located in the upper third of the stomach^[2,3]^. However, anastomotic leakage (AL) remains a serious and life-threatening complication following gastrectomy^[4,5]^, often necessitating additional treatment and extended hospitalization. Despite advancements in surgical techniques and postoperative care, the incidence of AL following proximal or total gastrectomy remains high (1.7–15%)^[6-8]^, significantly higher than that following distal gastrectomy. AL not only increases patient mortality but also prolongs hospital stays, escalates medical costs and diminishes the overall quality of patients’ lives^[9]^.

Currently, effective treatments for AL following gastrectomy are limited, rendering the early identification and prediction of AL critical for improving patient outcomes^[10,11]^. Traditional risk factors for AL include patient-related factors such as age, comorbidities and nutritional status, as well as surgical factors such as anastomosis, surgical technique and perioperative management^[8,12]^. AL remains an unmet medical need due to the limitations of current prediction models. While recent studies have proposed risk stratification tools, their clinical utility is constrained by modest accuracy and reliance on retrospective data^[12–14]^.

Machine learning has become an indispensable tool in modern medical research and practice^[15,16]^. It significantly enhances diagnostic accuracy and treatment efficiency, while also playing a crucial role in advancing the frontiers of medical research^[17]^. As computational technology continues to advance and applications expand, the role of machine learning in the medical field is expected to become increasingly central, potentially evolving into an important tool for supporting medical decision-making^[16,18,19]^. In our previous study, a predictive model was developed based on machine learning techniques, which demonstrated superior performance compared with existing models reported by retrospective studies^[20]^. The aim of the present study was to investigate the clinical utility of this real-time prediction model, which aims to assess the model’s performance, reliability and clinical utility across diverse patient populations and healthcare settings. This study has been reported in line with the STROCSS 2025 criteria^[21]^.

**HIGHLIGHTS**

- This prospective multicenter study (*n* = 512) evaluated a real-time machine learning model for predicting anastomotic leakage (AL) in gastric adenocarcinoma patients undergoing esophagogastric/esophagojejunal reconstruction.
- The model demonstrated an AUC of 0.780, sensitivity of 0.769, and NPV of 0.990, effectively stratifying high-risk (5.4% AL rate) versus low-risk groups (1.0%, *P* = 0.027).
- Post hoc analysis revealed the model’s potential to safely exempt approximately 35% of cases from intensive postoperative surveillance.

## Materials and methods

### Study population

**Figure 1.**
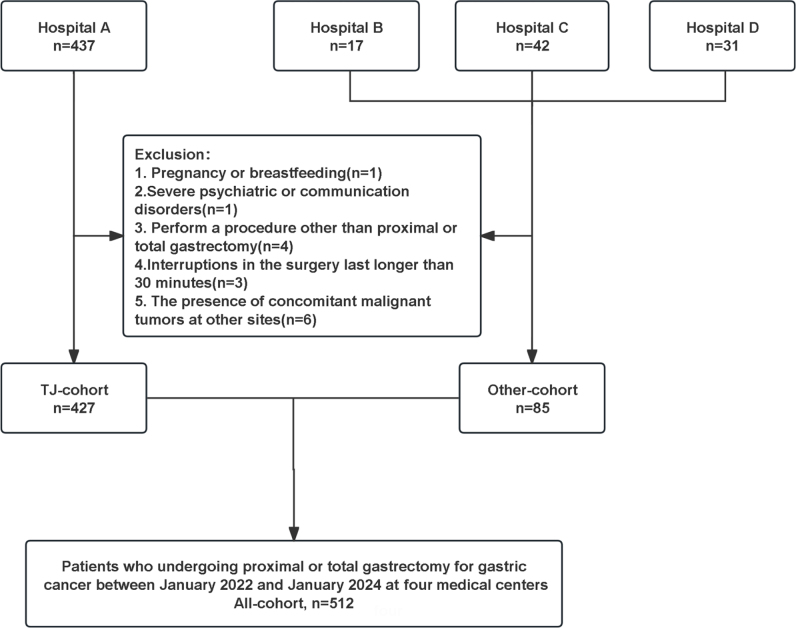
Schematic of patient screening process.

The aim of this prospective multicenter study was to validate a previously used predictive model. Consecutive patients who underwent gastric cancer surgery between January 2022 and January 2024 at four Chinese medical centers, including Hospital A, Hospital B, Hospital C and Hospital D were included in the present study. The inclusion criteria were as follows: Age, 18–85 years, preoperative pathological confirmation of primary gastric adenocarcinoma, the American Society of Anesthesiologists grade III or lower, and planned performance of proximal gastrectomy or total gastrectomy. Patients with stage I, II, resectable stage III disease, and intraoperative findings showed a solitary resectable intra-abdominal metastasis were enrolled, with D2 + CME (D2 lymphadenectomy plus complete mesogastric excision) or standard D2 resection serving as the definitive surgical approach. Informed consent was obtained from all participants. Exclusion criteria included pregnancy or breastfeeding, severe psychiatric or communication disorders, an intraoperative decision to perform a procedure other than proximal or total gastrectomy (including combined thoracotomy), interruptions in the surgery that lasted longer than 30 min for any reason, and the presence of concomitant malignant tumors at other sites. Patients from Hospital A were classified as the TJ cohort, while patients from the other centers were grouped into the Other cohort. The patient screening process is illustrated in Figure 1. This study was approved by the local institutional review board and was registered as a clinical trial.

### AL risk calculation

**Figure 2.**
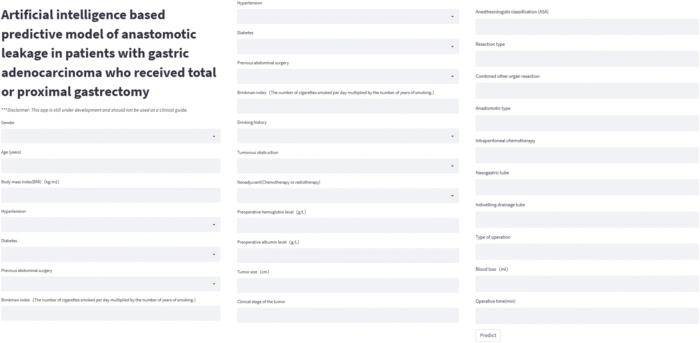
Schematic diagram of the online prediction website.

All patient-related data were recorded and simple min–max normalization was conducted to transform the continuous variables to values within the range of 0 and 1 using the previous parameters; dichotomous variables were represented by 0 or 1^[20]^. The surgeons used a previously published online tool with random forest kernel (https://gasal.21cloudbox.com/; Fig. 2) to output AL risk using patients’ preoperative and intraoperative clinical data. Specifically, in the previous publication, we developed a random forest model (n_estimators = 100, max_depth = 4, min_samples_split = 2) using routinely collected clinical variables from 1660 patients, which demonstrated strong discriminative ability (AUC = 0.90) after data preprocessing with downsampling and min–max normalization, along with hyperparameter optimization via grid search.

### AL definition

The diagnosis of AL primarily relies on the patient’s clinical symptoms, and laboratory and imaging examinations. A diagnosis of AL was confirmed if any of the following criteria were diagnosed: (i) Abnormal drainage fluid: The presence of digestive fluids or food residue in the abdominal drainage or puncture tube; (ii) when the patient presented with fever, abdominal pain and peritonitis, the diagnosis of AL was suspected, and upper gastrointestinal contrast and CT examination were conducted. The diagnosis of AL is confirmed in the following scenarios: Leakage of water-soluble contrast agent outside the gastrointestinal tract following oral administration and detection of air bubbles, and encapsulated fluid around the anastomotic site; (iii) Reoperation for any reason due to interruption of anastomotic integrity was also considered as AL^[4]^. In addition, for cases where the diagnosis of AL remained uncertain, a review panel of two senior gastrointestinal surgeons deliberated and determined the classification.

### Statistical methods

Statistical methods were analyzed using χ^2^ tests for categorical variables and *t*-tests for continuous variables. The comparison between AUCs was conducted using the Delong test. All statistical analysis was performed in Python (version 3.12) and R (version 4.3.3). All statistical tests were two-tailed. *P* < 0.05 was considered to indicate a statistically significant difference.

## Results

### Patient characteristics

A total of 512 consecutive eligible patients were included in the final analysis, and their characteristics are presented in Table 1. The mean age of the participants was 62.82 ± 9.23 years, with 73.24% (375/512) being male. The mean body mass index was 22.57 ± 3.45 kg/m^2^. The laparoscopic technique was used in 510 patients (99.61%), with only 2 patients (0.39%) requiring concurrent removal of other organs. In addition, 85 patients (16.60%) received intraoperative intraperitoneal chemotherapy to reduce the risk of recurrence. The comparison between the TJ cohort and the other cohort is shown in Supplementary Table 1, Available at, http://links.lww.com/JS9/E682.

**Table 1:** Patient characteristics

Variables	Total (*n* = 512)
Demographic characteristics	
Age (years), mean ± SD	62.82 ± 9.23
Body mass index (kg/m^2^), mean ± SD	22.57 ± 3.45
Male, *n* (%)	375 (73.24)
Comorbidities	
Brinkman index, mean ± SD	209.02 ± 810.58
Hypertension, *n* (%)	161 (31.45)
Diabetes, *n* (%)	49 (9.57)
American Society of Anesthesiologists classification, *n* (%)	
1	12 (2.34)
2	337 (65.82)
3	163 (31.84)
Previous abdominal surgery, *n* (%)	73 (14.26)
Drinking history, *n* (%)	77 (15.04)
Laboratory variables	
Hemoglobin level (g/L), mean ± SD	117.28 ± 23.12
Albumin level (g/L), mean ± SD	38.71 ± 3.81
Tumor-associated variables	
Tumor size (cm), mean ± SD	4.02 ± 2.14
Tumorous obstruction, *n* (%)	71 (13.87)
Neoadjuvant chemotherapy or radiotherapy, *n* (%)	24 (4.69)
Clinical stage of the tumor, *n* (%)	
1	115 (22.46)
2	173 (33.79)
3	215 (41.99)
4	9 (1.76)
Intraoperative variables	
Total gastrectomy, *n* (%)	413 (80.66)
Laparoscopic surgery, n (%)	510 (99.61)
Esophagojejunostomy, *n* (%)	415 (81.05)
Combined other organ resection, *n* (%)	2 (0.39)
Intraperitoneal chemotherapy, *n* (%)	185 (36.13)
Nasogastric tube, *n* (%)	482 (94.14)
Indwelling drainage tube, n (%)	507 (99.02)
Blood loss (mL), mean ± SD	69.49 ± 128.34
Operative time (min), mean ± SD	278.68 ± 91.94
Outcomes	
Anastomotic leakage, *n* (%)	13 (2.54)

SD, standard deviation.

### Characteristics

**Figure 3.**
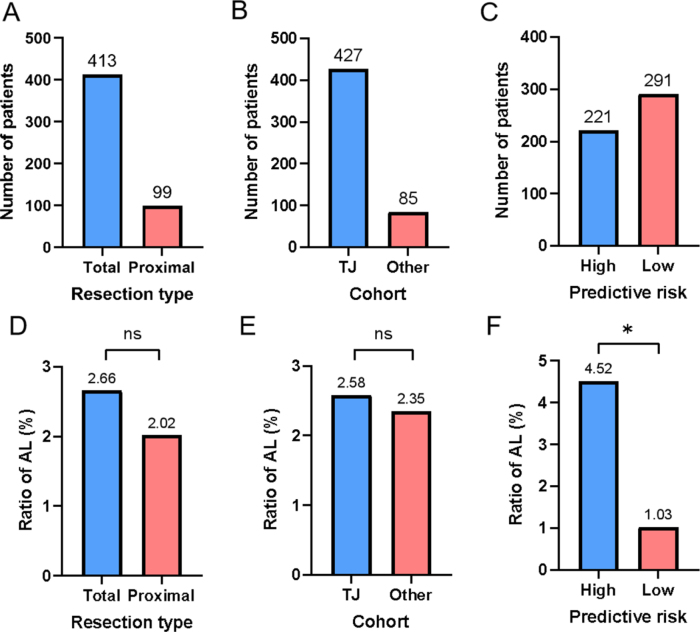
Patient information and incidence of AL. (A, D) Number of patients and ratio of AL for total and proximal gastrectomy. (B) Number of patients in the TJ and Other cohorts. (C) Number of high and low risk of AL calculated by our model for all participants. (E) Comparison of AL rate between the TJ and Other cohorts. (F) Incidence of AL in high- and low-risk participants. TJ, Hospital A; AL, Anastomotic leakage.

AL was developed by 13/512 participants (2.54%). Among the 512 patients, 413 underwent total gastrectomy, while the remaining 99 received proximal gastrectomy (Fig. 3A). The AL rate was 2.66% (11/413) for total gastrectomy and 2.02% (2/99) for proximal gastrectomy, which was similar to the finding of our previous study^[20]^. There was no statistically significant difference between the two groups (Fig. 3D). In addition, the AL rates between the TJ and Other cohorts were also not statistically significant (Figs. 3B and E).

### Model performance

**Figure 4.**
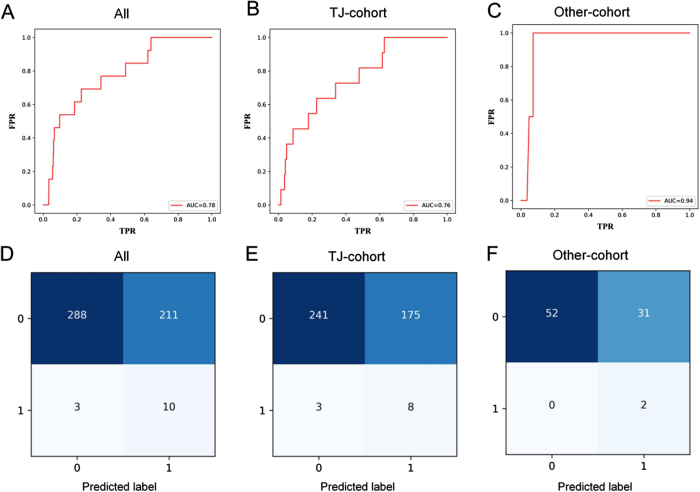
Receiver operating characteristic curve and confusion matrix for overall, TJ and Other cohort. TJ, Hospital A; FPR, False Positive Rate; TPR, True Positive Rate.

**Figure 5.**
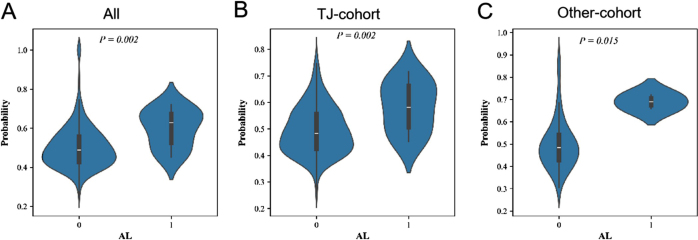
Comparison of predicted probability of AL between patients with and without AL in the overall, TJ and Other cohorts. TJ, Hospital A; AL, Anastomotic leakage.

The receiver operating characteristic (ROC) curve of the model for predicting AL in the overall dataset (TJ and Other cohorts) is shown in Figure 4A, with an area under the curve (AUC) of 0.78. Given the potential impact of AL’s low incidence on model performance, we performed bootstrap validation to rigorously assess predictive capability, confirming robust performance across resampling iterations (Supplementary Figure 1, Available at, http://links.lww.com/JS9/E681). The confusion matrix obtained from applying our previous machine learning model showed good performance with a sensitivity of 0.769, specificity of 0.577, negative predictive value (NPV) of 0.990, positive predictive value(PPV) of 0.045 and accuracy of 0.582 (Fig. 4D). To further evaluate the PPV performance, we conducted comprehensive sampling analyses including oversampling and undersampling techniques, which demonstrated that PPV could improve to 0.18–0.22 in artificially balanced datasets, confirming that the modest PPV in our primary analysis primarily reflects the low baseline incidence of AL (2.54%) rather than model limitations(Supplementary Figure 2, Available at, http://links.lww.com/JS9/E681). Of these 512 patients, 221 were classified as high-risk, and the other 291 as low-risk (Fig. 3C). The AL rate was significantly higher in the high-risk group compared with the low-risk group (10/221 vs. 3/291; *P* = 0.027; Figure 3F), which indicated a good risk stratification ability of the model. In addition, the comparison of calculated risk values showed that the patients with AL had a higher risk than those without AL (*P* = 0.002; Figure 5A).

### Model performance in the TJ and Other cohorts

In addition to overall participants, the model was also assessed in the TJ and Other cohorts, separately. The ROCs of TJ and Other cohorts showed good discernment with AUCs of 0.760 and 0.940 (Figs. 4B and C). Upon validation, the confusion matrices for the TJ and Other cohorts were obtained (Figs. 4E and F). The data indicated that the model performed with a sensitivity of 0.727, specificity of 0.579, NPV of 0.988, PPV of 0.044 and accuracy of 0.583 in the TJ cohort. In the Other cohort, the model performed with a sensitivity of 1.00, specificity of 0.627, NPV of 1.000, PPV of 0.061 and accuracy of 0.635. After comparing the probability calculated by the model, AL patients were found to have a higher risk of developing AL that those without AL in both the TJ and Other cohorts (Figs. 5B and C).

### Feature importances

**Figure 6.**
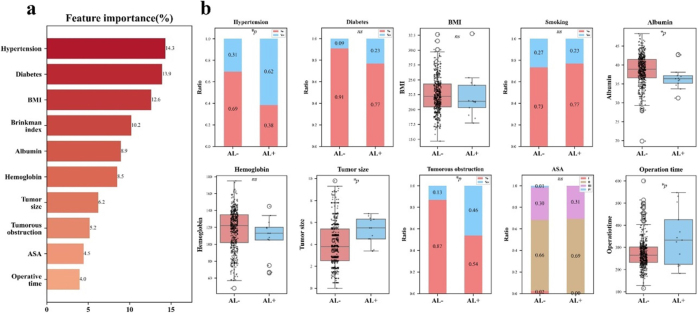
Predictive variable analysis. (A) Variable importance ranking from the model. (B) Clinical correlation between identified variables and actual AL occurrence(*P < 0.05,). TJ, Hospital A; AL, Anastomotic leakage.

During the initial model development phase, hypertension, diabetes mellitus, BMI, Brinkman index, albumin level, hemoglobin level, tumor size, tumor obstruction, ASA classification, and operative time were identified as important predictive factors (Fig. 6a). To evaluate the robustness of these variables, we further assessed their clinical correlations with AL occurrence. The analysis revealed that hypertension, albumin levels, tumor size, tumor obstruction, and operative time demonstrated statistically significant associations (*P* < 0.05). While diabetes mellitus, BMI, and hemoglobin levels did not reach statistical significance, they nevertheless exhibited clinically relevant trends (Fig. 6b).

### Post Hoc analysis

**Figure 7.**
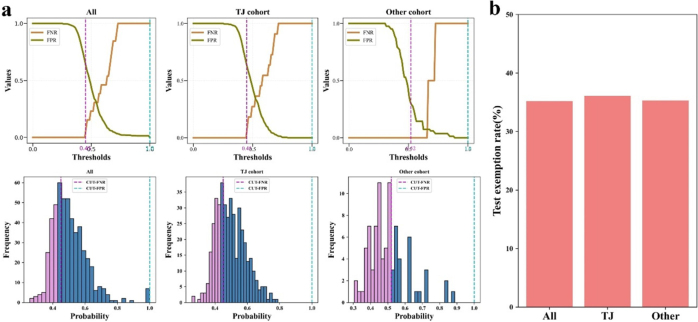
Model performance thresholds and clinical implications. (A) False-positive and false-negative rates as functions of the prediction threshold, showing complete elimination of false negatives at risk scores <0.45. (B) Theoretical proportion of patients eligible for exemption from intensive postoperative monitoring.

To further evaluate clinical utility of the model, we performed post hoc analyses. The false-positive/false-negative rate analysis revealed a critical threshold at 0.45, below which no false negatives occurred (Fig. 7a). This threshold reliably identified 35.2% of patients (180/512) as low-risk who could safely forgo intensive monitoring. Notably, this pattern remained consistent across both the TJ cohort (36.1%, 154/427) and other cohort (35.3%, 30/85) (Fig. 7a, 7b). Furthermore, subgroup analyses demonstrated superior predictive performance of our model compared to single-variable predictions, highlighting its enhanced predictive capability (Supplementary Figure 3, Available at, http://links.lww.com/JS9/E681). These findings demonstrate that our predictive model can reliably identify approximately one-third of patients who will not develop AL (NPV = 100%). This capability enables clinicians to safely implement accelerated recovery protocols for these low-risk patients while potentially reducing hospitalization costs through avoided diagnostic tests and shortened stays, offering meaningful clinical and economic advantages over current standard practices.

## Discussion

Gastric adenocarcinoma is the most prevalent malignancy in the upper gastrointestinal tract^[1]^, with surgical resection serving as the primary treatment method. Although surgery has demonstrated therapeutic efficacy, serious complications such as AL still occur postoperatively^[22–24]^. AL allows digestive fluids to leak into the abdominal cavity, potentially leading to peritonitis, abdominal abscess, bleeding and infectious shock, all of which can endanger the patient’s life. Thus, timely intervention is crucial upon AL occurrence, with treatments ranging from fasting and drainage to secondary surgery, depending on severity^[25]^. Preventing AL following gastric cancer surgery remains a significant challenge in gastrointestinal surgery.

Postoperative AL is multifactorial, and reliable risk prediction tools for gastrointestinal leakage following gastric cancer resection are still lacking, making it difficult to assess or quantify AL risk. Recently, machine learning-based models have shown strong potential in predicting adverse outcomes^[15,18,26–29]^. For example, Ken Porche *et al* developed an ML model that accurately predicts urinary retention risk following lumbar spine surgery, validated through prospective studies^[29]^. Similarly, Charlie Saillard *et al*^[30]^ demonstrated the effectiveness of MSIntuit, an AI-based tool for detecting microsatellite instability in colorectal cancer. Our previous work involved machine learning algorithms to predict esophagogastric and esophagojejunal AL with high accuracy^[20]^. With the advancement of predictive models for disease risk, several models have been developed to assess AL in gastric cancer and identifying key risk factors to mitigate AL^[12,14,31]^. However, some limitations of these models, such as reliance on single-center, retrospective data, and the absence of large and multicenter prospective validations create challenges for clinical application. These factors along with variations in sample characteristics, geographic region, and time period limit the generalizability and reliability of existing models^[32]^.

Our earlier risk prediction models also suffered from similar limitations, including biases inherent in single-center retrospective data. To address this, the current study included prospective validation from four tertiary medical centers. In this prospective validation study, the incidence of AL was 2.54% (13/512) following proximal and total gastrectomy, closely matching the 2.17% (36/1660) observed in the model development dataset and published Asian center researches^[12,14,33]^. Previously, using retrospective data from 1660 patients between 2010 and 2019, the model performed with an AUC of 0.900 in the test cohort. The model’s AUC was 0.780, indicating a slightly lower performance than the initial validation. In this prospective validation, the model’s specificity was 0.577 (previously 0.822), the sensitivity was 0.769 (previously 0.818), the NPV was 0.990 (previously 0.992), the PPV was 0.045 (previously 0.137) and the accuracy was 0.582 (previously 0.822). While some performance metrics showed expected variation in prospective validation, the model maintains distinct advantages over conventional prediction tools. It demonstrates superior discrimination compared to widely used nomograms like Tu’s score^[12]^ (AUC 0.78 vs 0.65-0.70), while offering real-time risk calculation through our web-based interface, particularly valuable for intraoperative decision-making. Most significantly, the model’s unique capacity to definitively identify ~ 35% of patients as low-risk (NPV = 100%) enables safe reduction of postoperative monitoring intensity, an advantage unavailable in traditional scoring systems. Although the modest PPV (4.5%) reflects the inherent challenge of predicting rare events, the exceptional NPV (99.0%) provides safety for excluding AL in low-risk cases. Together, these features represent a clinically meaningful advance over existing prediction paradigms.

The predictive factors incorporated in our model ranging from objective clinical variables (e.g., serum albumin levels, operative duration) to standardized assessments (e.g., ASA classification) were rigorously selected based on their established associations with AL in prior studies^[4,8]^. Variable importance analysis confirmed the robustness of these predictors, enabling their dual application for risk stratification and targeted clinical management. For high-risk patients, the model may be helpful in guiding controllable factors modification, intraoperative decision-making and postoperative management, such as, preoperative albumin supplementation, tight glycemic control in diabetics and optimized blood pressure management, selective staple-line reinforcement and protocolized imaging or delayed feeding. Importantly, the model’s exceptional NPV (100% for risk scores <0.45) identifies ~ 35% of patients who can safely avoid intensive monitoring, enabling reallocation of clinical resources without compromising patient safety. This paradigm shift transforms passive risk prediction into active, individualized prevention strategies. Furthermore, these findings provide foundational work for future dynamically updated or EHR-integrated AI systems.

However, the present study had several limitations. Four Chinese medical centers were used as the validation set, but data from other countries may provide different results. While multicenter inclusion strengthens generalizability, the TJ-dominated sample may still limit extrapolation to vastly different healthcare settings. We mitigated this through prospective validation and external center testing, but recommend further validation in balanced multinational cohorts. Despite the performance at the high sensitivity, the PPV of 4–5% needs to be improved. In addition, differences in medical equipment, resources, regional treatment preferences or surgical procedures, patient populations and data collection methods between centers may have introduced heterogeneity, potentially affecting the comparability of the findings.

## Conclusions

By achieving high sensitivity comparable to the ground truth, while excluding nearly half of the non-AL cases, the model (available at https://gasal.21cloudbox.com/) offers effective risk stratification for AL in patients with gastric adenocarcinoma undergoing esophagogastrostomy or esophagojejunostomy.

## Data Availability

Due to ethical restrictions, the raw data cannot be made publicly available. However, deidentified data may be obtained from the corresponding author upon reasonable request.

## Research Registration Unique Identifying Number (UIN)

Clinicaltrials: registered as a clinical trials (no. NCT05646290).

## Data sharing statement

The data and code used during the study is available from the first and corresponding authors on reasonable request.
